# Nutritional Myeloneuropathy Secondary to Thiamine Deficiency: A Case Report

**DOI:** 10.7759/cureus.84788

**Published:** 2025-05-25

**Authors:** Yazan Mazen Yaser Saidismail, Masa Alashkar, Mohamed Nasr Elsaid, Karim Abdalbari, Aliaa Al Chaar, Ahmed Darweesh, Ahmad Alli Alshouraa, Shahid Hamid, Leena Abdelrahman

**Affiliations:** 1 General Practice, University of Sharjah, Sharjah, ARE; 2 Internal Medicine, Dubai Hospital, Dubai Health, Dubai, ARE; 3 College of Medicine, Mohammed Bin Rashid University of Medicine and Health Sciences, Dubai, ARE; 4 Internal Medicine, Kuwait Hospital Sharjah, Emirates Health Services, Sharjah, ARE

**Keywords:** dry beriberi, lower limb weakness, peripheral vision loss, sleeve gastrectomy, thiamine deficiency

## Abstract

Thiamine (vitamin B1) deficiency can occur secondary to malnutrition, which is associated with multiple etiologies, from imbalanced dietary habits to significant gastrointestinal nutritional losses such as recurrent vomiting or diarrhea. Thiamine deficiency manifests as dry or wet beriberi, Wernicke encephalopathy, or Korsakoff syndrome. We present a 26-year-old lady, three months post-laparoscopic sleeve gastrectomy with a complicated postoperative course due to poor oral intake and recurrent vomiting, who presented with bilateral limb weakness and vision loss of five days duration. Physical examination was significant for decreased power strength in her lower limbs relative to the upper limbs, and absent reflexes in her lower limbs. She was extensively tested for different disease-causing neuropathies, including lumbar puncture, imaging studies of the brain and spine regions, nerve conduction studies, and autoimmune disorders. Investigations revealed significantly low thiamine levels compared to other vitamins and minerals. She was managed with vitamin supplementation and aggressive physiotherapy. This case emphasizes the importance of considering nutritional deficiencies as a differential diagnosis for neuropathies, especially with certain risk factors such as poor nutritional intake or gastrointestinal operations or losses.

## Introduction

Thiamine (vitamin B1) is a vital nutrient that plays a crucial role in converting the energy from food into fuel for the brain, nerves, and the heart [1]. Thiamine is primarily absorbed in the small intestine, where it is transported into the bloodstream for use by various tissues in the body. Thiamine deficiency is a serious complication of bariatric surgery that can result in permanent and severe consequences if not diagnosed and treated promptly. The risk of developing thiamine deficiency is rare in the general population, making it easy for healthcare providers, particularly those unfamiliar with bariatric surgery, to overlook or misdiagnose [2,3]. Certain risk factors include inadequate intake or impaired absorption, which occur in conditions like chronic alcoholism and malnutrition [3-5]. 

Other causes involve increased loss of thiamine, as seen in hyperemesis gravidarum and diarrhea. Increased utilization of thiamine occurs in pregnancy and hyperthyroidism, which can result in deficiency as well [4]. Postoperative nausea and vomiting, which can be frequent and recurrent, is another possible mechanism for thiamine deficiency after bariatric surgery. This complication may be associated with the patient’s eating behaviors or surgical complications, such as stenosis or small bowel obstruction. Postoperative reduction in energy intake may require patients to take additional vitamin and mineral supplementation. Deficiencies may result from not adhering to the prescribed supplements, including thiamine deficiency [2]. A recent study review was conducted and revealed that 27% of patients who underwent bariatric surgeries experience vitamin B1 deficiency. As a result, lifelong supplementation of vitamin B1 should be recommended for these patients following surgery [6].

Thiamine deficiency can lead to a range of symptoms, including fatigue, irritability, muscle weakness, and confusion. In severe cases, it can cause neurological complications such as peripheral neuropathy, ataxia, and Wernicke-Korsakoff syndrome, a condition that affects memory and coordination [1,5]. In severe cases, thiamine deficiency can present as either wet or dry beriberi. Wet beriberi primarily affects the cardiovascular system, leading to symptoms like edema, shortness of breath, and heart failure. Dry beriberi, on the other hand, impacts the nervous system, causing symptoms such as muscle weakness, neuropathy, and difficulty with coordination [1,5]. We report a case of a young woman presenting with nutritional myeloneuropathy post-sleeve gastrectomy. Raising awareness among healthcare providers in the community about the heightened risk of thiamine deficiency in bariatric patients, along with its causes, symptoms, and treatment, is crucial, as it can greatly enhance patient outcomes and quality of life.

## Case presentation

A 26-year-old woman, known case of morbid obesity post-laparoscopic sleeve gastrectomy on 21st of August 2024, and multiple hospital admissions post-laparoscopic sleeve gastrectomy due to recurrent nausea and vomiting, poor oral intake, and abdominal pain, presented to the emergency department with bilateral lower limb weakness of five days duration. This was associated with peripheral vision loss, inability to maintain eye contact, and altered mental status. Her vitals were within the normal limits. Neurological examination revealed a Glasgow Coma Scale of 15/15 with altered mental state, power of 4+/5 in all upper limb muscle groups, and lower limb power of 3/5 proximally and 4-/5 distally. She had brisk deep tendon reflexes in both upper limbs, absent reflexes, and downgoing plantar reflexes in both lower limbs. She had a normal sensory assessment in her upper and lower limbs. Slit lamp exam revealed abnormal bilateral ocular movements.

**Figure 1:**
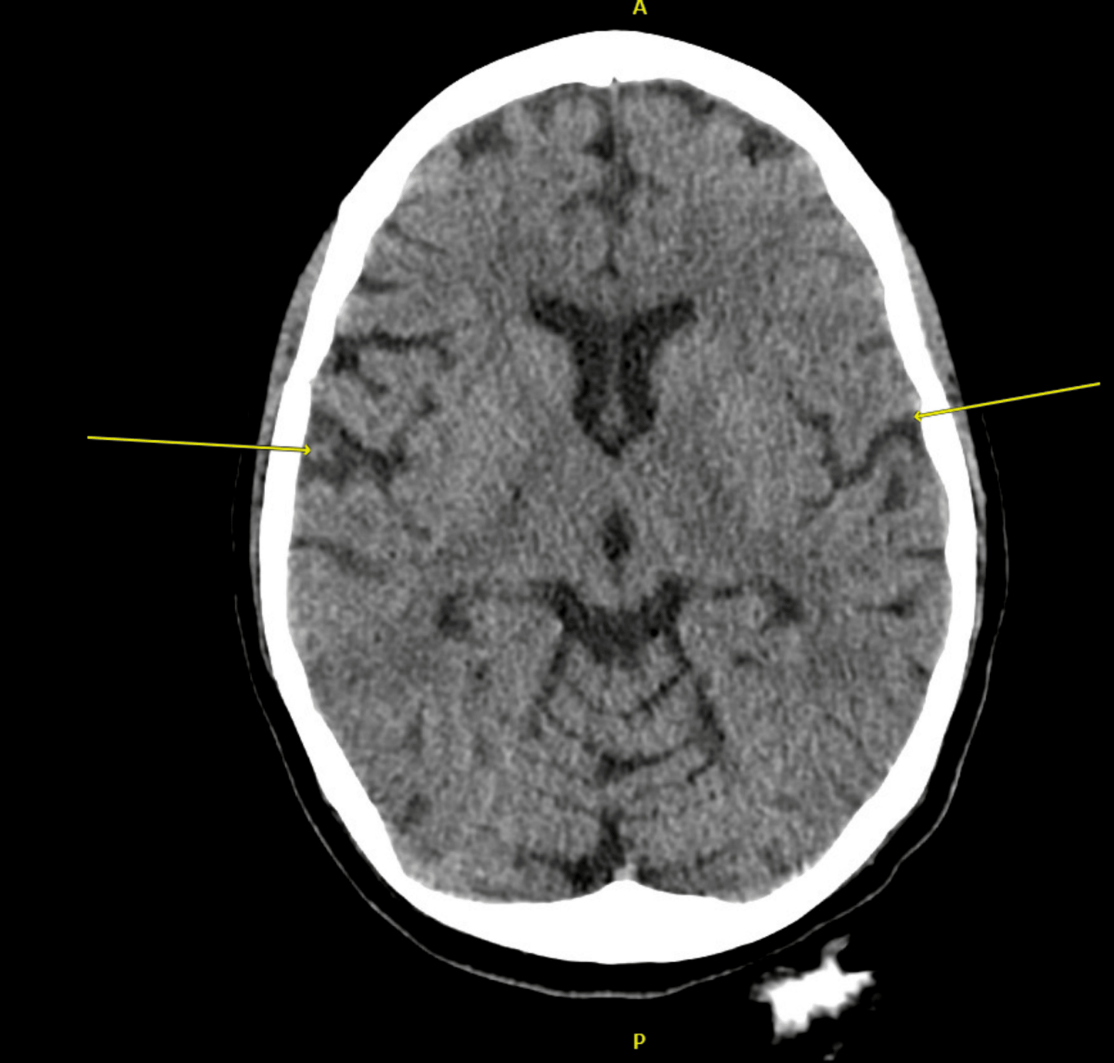
CT scan of the brain without contrast, showing mild enlargement of the lateral ventricle and diffuse cortical atrophy, characterized by mild widening of lateral fissure (yellow arrows)

**Figure 2:**
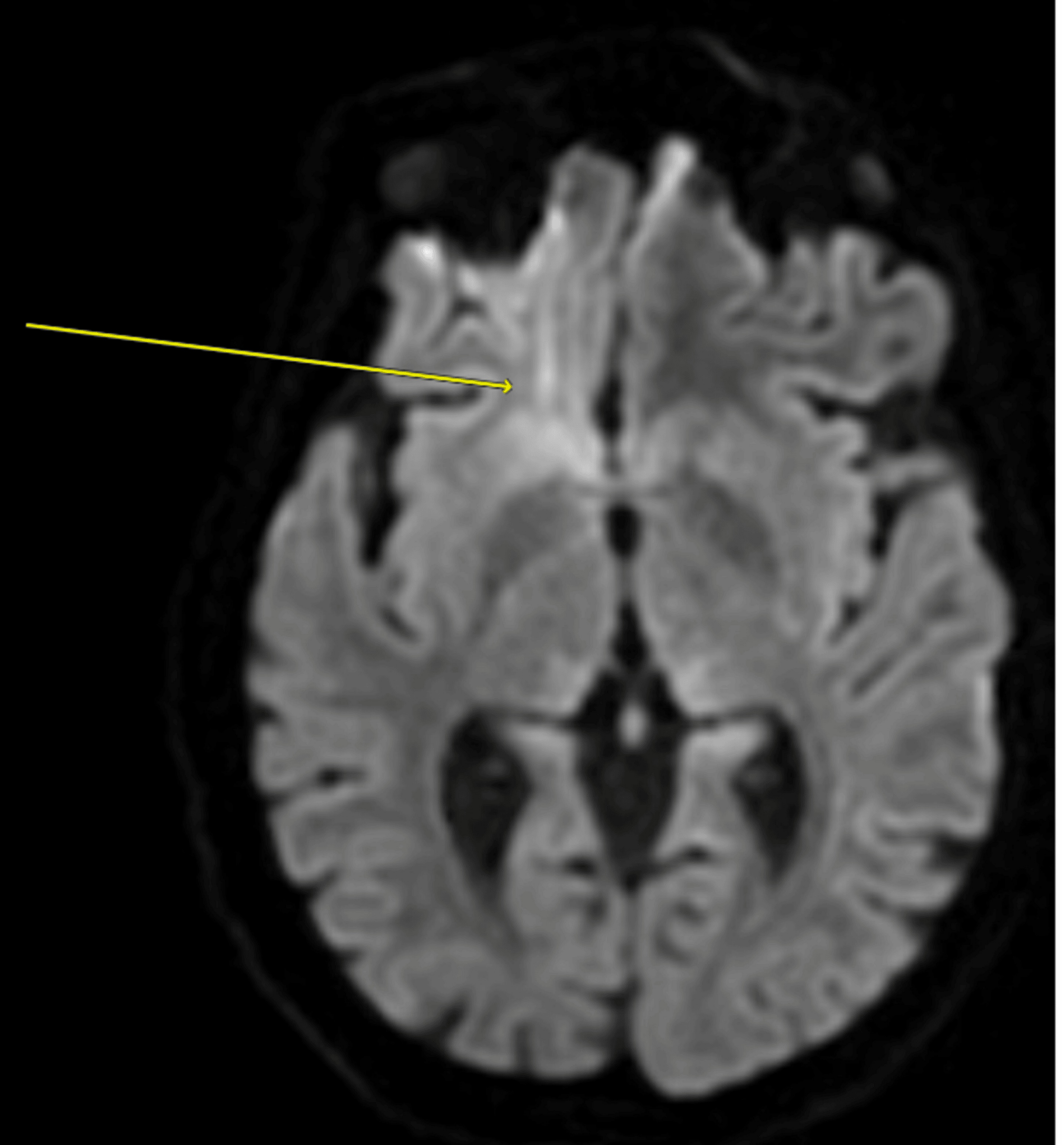
MRI scan of the brain with contrast, showing focal hyperintense signals in the right frontal subcortical region (yellow arrow) in the DWI sequence, which may suggest cerebral edema

Computed tomography (CT) scan of the brain without contrast revealed early atrophic changes of the brain that are more than expected for the patient's age. This was followed by further imaging tests, including a magnetic resonance imaging (MRI) scan of the brain with contrast. This test was significant for focal hyperintense signals in the right frontal subcortical region in the diffusion-weighted imaging (DWI) sequence. An MRI scan of the spine was done, but was unremarkable with no evidence of demyelination appreciated. Figures 1-2 show the findings in the CT and MRI scans of the brain, respectively.

Lumbar puncture was done for her. Cerebrospinal fluid (CSF) analysis results were only significant for elevated CSF proteins (54 mg/dL; normal 15045 mg/dL) with no white blood cells seen. CSF meningitis and encephalitis panels, oligoclonal bands, aquaporin-4 antibodies (neuromyelitis optica or NMO-IgG), myelin oligodendrocyte glycoprotein (MOG) antibodies, and angiotensin-converting enzyme levels were all negative. Blood tests were significant for abnormally low vitamin B1 levels. Copper, zinc, magnesium, and ammonia levels were within normal ranges. Vitamins B9 (folic acid) and B12 were elevated secondary to over-supplementation from her previous admissions. Autoimmune antibodies - including vasculitis antibodies - and lupus anticoagulant tests were negative. TABLE 1 shows a timeline of the different vitamins and minerals that were assessed. Nerve conduction studies were normal as well.

**Table 1:** Timeline of the patient's vitamins and minerals levels.

Micronutrient	Results				Reference range
	July 2024	October 2024	November 2024	December 2024	
Vitamin B1 (thiamine)	35	15 (L)	9 (L)	57	28-85 mcg/L
Vitamin B2 (riboflavin)		202	144 (L)	189	180-295 mcg/L
Vitamin B3 (niacin)			17.3		8-100 mcg/L
Vitamin B6 (pyridoxine)	8.3	3.3 (L)	4.8 (L)	15.4	5-50 mcg/L
Vitamin A (retinol)		0.37			0.3-0.6 mg/L
Selenium		56	54 (L)		55-103 mcg/L

The patient was diagnosed with nutritional myeloneuropathy secondary to thiamine deficiency due to her recurrent loss of nutrients by vomiting and decreased nutritional intake. The patient was treated with dietary modifications, regular intravenous thiamine infusion followed by regular intramuscular administration of vitamins B1, B6, and B12, increased protein, vitamin, and mineral intake, and physiotherapy. Vitamins B1, B6, and B12 were switched to oral tablets later. With vitamin supplementation and physiotherapy, her weakness has significantly improved, and her vitamin B1 levels returned to normal, as noted in Table 1.

## Discussion

Thiamine is essential for adenosine triphosphate (ATP) production. It is a critical, rate-limiting cofactor to multiple enzymes involved in this process [7]. The early symptoms of thiamine deficiency are non-specific and may be easily attributed to any number of disease processes. Despite the critical role thiamine plays in cellular metabolism, deficiency often manifests in vague and nonspecific early symptoms, making diagnosis challenging. Unrelenting or uncharacteristic fatigue, irritability, and mood lability [7], and a sense of mental fuzziness and subtle decrements in memory are often reported, as well as heaviness and weakness of the legs [2]. Early symptoms of thiamine deficiency are so vague; therefore, the clinical descriptions tend to focus on its later-stage manifestations, such as wet and dry Beriberi, as well as Wernicke’s encephalopathy and Korsakoff’s syndrome [7]. A post-mortem investigation of 131 cases of Wernicke’s encephalopathy found that 80% were missed during life, likely because only 16% presented with the classic triad. Around 44% had one or two of the three symptoms, while 19% had none at all [8].

Thiamine deficiency is a known complication following bariatric surgeries, particularly sleeve gastrectomies, with numerous cases reported in the literature [6,9]. Despite being highly effective for weight loss, bariatric surgeries can have unintended consequences on nutrient absorption [10]. These surgeries often lead to achlorhydria, or insufficient gastric acid production, which impairs the absorption of water-soluble vitamins like vitamin B1. In addition, some patients experience persistent vomiting after surgery, further contributing to the loss of these vitamins. When combined with the increased metabolic demands during the postoperative phase, these factors help explain the occurrence of thiamine deficiency in these patients [9,11]. Most patients undergoing bariatric surgery have low levels of nutrients before the procedure, and these levels are reduced further after the procedure, as reported in the literature [10]. They are at an increased risk of developing thiamine deficiency, particularly after an episode of intractable vomiting [2].

Documented examples of thiamine deficiency-induced neuropathy are consistent with the patient's clinical presentation, which is characterized by marked sensory and motor impairments. One case report, for example, described a patient who experienced dry beriberi following sleeve gastrectomy and who had neurological symptoms that were linked to fast weight loss and insufficient thiamine intake [12]. Another case report documented the occurrence of Wernicke's encephalopathy in a patient following sleeve gastrectomy, highlighting persistent postoperative vomiting as a significant contributing factor to the development of thiamine deficiency [13].

Classical neuroimaging findings of thiamine deficiency on MRI brain scans are of Wernicke’s encephalopathy - cerebral edema (both cytotoxic and vasogenic) is commonly encountered. Typically, lesions are bilateral, and commonly affected areas include the thalami and mammillary bodies. Atypical regions include the cerebellum and cerebral cortices (especially the frontal and parietal cortices in the latter) [14]. According to Dhir et al., thiamine deficiency may affect different parts of the brain in adults compared to the pediatric population. In addition to the areas affected earlier, adults may have the brainstem and hypothalamus affected. Children tend to have the mammillary bodies involved, as well as the basal ganglia and the frontal lobes [15]. In this study, the patient had unilateral involvement of the right frontal subcortical area.

The pattern of atypical imaging findings in thiamine deficiency is common and may be associated with the underlying etiology. A recent monocentric retrospective study conducted in France reported that around a third of cases had non-specific MRI findings, and one-third were considered normal [16]. It seems that the distribution of atypical lesions is observed more with patients who do not have chronic or regular alcohol intake [14].

Despite having clinical neuropathy, this patient's nerve conduction studies were normal. A previous case study by Murate et al. discussed a middle-aged lady who complained of lower limb muscle weakness and pain that improved after thiamine replacement therapy, supporting a diagnosis of thiamine deficiency neuropathy. Her nerve conduction study had normal motor conduction velocities and compound muscle action potential amplitude, but revealed mild generalized large fiber sensory axonopathy. This patient also underwent needle electromyography that showed findings associated with acute motor axonopathy. Other studies revealed that thiamine deficiency manifests with motor nerve conduction study abnormalities [17]. This may support the hypothesis that thiamine deficiency can manifest variably in different neurophysiological studies between patients; a combination of history and physical examination, along with the different investigations available, helps in diagnosing thiamine deficiency.

This patient had a preoperative vitamin B1 level of 35 mcg/L, slightly above the lower normal baseline level of vitamin B1 (28-85 mcg/L), making her at a high risk of developing thiamine deficiency. Her postoperative course was complicated with multiple hospital admissions due to poor oral intake and frequent nausea and vomiting. Although vitamin B complex supplements were administered to her, her vitamin B1 levels continued to drop to 15 mcg/L, then to 9 mcg/L. On the contrary, her vitamins B9 and B12 were markedly elevated due to over-supplementation. It was noted that after regular intravenous thiamine replacement, in addition to the intramuscular vitamin B complexes, her vitamin B1 levels reached 57 mcg/L, higher than her preoperative level. This reinforces the need for vigilant supplementation post-surgery. Micronutrient supplements, including thiamine, are routinely recommended post-bariatric surgery to prevent deficiencies.

This case underscores the critical importance of routine postoperative nutritional surveillance and patient education. Regular monitoring of thiamine levels is essential, particularly in patients presenting with symptoms such as nausea, vomiting, or neurological deficits. Establishing standardized protocols for nutritional supplementation and ensuring adherence can significantly reduce the risk of such complications. Evidence in the literature consistently highlights the necessity of early identification and prompt management of thiamine deficiency to avert irreversible neurological sequelae [12].

## Conclusions

This case highlights the critical need to include thiamine (vitamin B1) deficiency in the differential diagnosis of neuropathies, especially for patients with risk factors such as gastrointestinal surgery, recurrent vomiting, or poor oral intake. The core finding was the diagnosis of nutritional myeloneuropathy in a 26-year-old female patient presenting with bilateral limb weakness and vision loss post-laparoscopic sleeve gastrectomy. Comprehensive diagnostic investigations identified markedly low thiamine levels, having excluded other potential neuropathic causes. Notably, the patient's weakness significantly improved, and thiamine levels normalized following supplementation and aggressive physiotherapy, underscoring the potential reversibility of such neurological deficits with prompt identification and treatment.

These findings have significant implications for healthcare providers. Firstly, a high index of suspicion for thiamine deficiency is essential in at-risk populations, as early symptoms can be vague and easily misattributed. Secondly, this case reinforces the necessity for routine postoperative nutritional surveillance and robust patient education, including vigilant monitoring of thiamine levels, particularly in individuals presenting with nausea, vomiting, or neurological symptoms. Adherence to standardized nutritional supplementation protocols is crucial to prevent these severe complications. Ultimately, early identification and swift management are paramount to averting irreversible neurological damage. Therefore, a comprehensive patient history and risk factor assessment are crucial, prompting consideration of thiamine deficiency once more common neuropathic causes are excluded, to ensure timely diagnosis and intervention.
